# Supplementary material for: The lymphatic filariasis treatment study landscape: A systematic review of study characteristics and the case for an individual participant data platform
Source: PLoS Negl Trop Dis. 2024 Jan 16;18(1):e0011882. doi: 10.1371/journal.pntd.0011882 (PMC10817204; doi:10.1371/journal.pntd.0011882)
Supplement: S1 Text — (DOCX) [file pntd.0011882.s001.docx]

**Database: Medline (Ovid MEDLINE)**

Database: Medline (Ovid MEDLINE Epub Ahead of Print, In-Process & Other Non-Indexed Citations, Ovid MEDLINE Daily and Ovid MEDLINE) 1946 to present

Search Strategy:

1 "lymphatic filarias?s".tw.

2 ("bancroftian filarias?s" or "brugia* filariasis" or "Malay* filarias?s" or elephantiasis or elephantiases or microfilar?emia or hydrocele or "filarial lymphoedema" or "filarial lymphedema" or "filarial lymphooedema").tw.

3 adenolymphangitis.tw.

4 (acute adj2 lymphangitis).tw.

5 dermatolymphangioadenitis.tw.

6 "B. pahangi".tw.

7 "circulating filarial antigen".tw.

8 brugiasis.tw.

9 "Wuchereria bancrofti".tw.

10 Brugia.tw.

11 "B. timori".tw.

12 "B. malayi".tw.

13 "W. bancrofti".tw.

14 "ADL attacks".tw.

15 ADLA.tw.

16 dermato-lymphangio-adenitis.tw.

17 Elephantiasis, Filarial/

18 Wuchereria bancrofti/

19 exp Brugia/

20 1 or 2 or 3 or 4 or 5 or 6 or 7 or 8 or 9 or 10 or 11 or 12 or 13 or 14 or 15 or 16 or 17 or 18 or 19

21 20

22 limit 21 to yr="1990 -Current"

**Database: Embase 1974 to present**

**Search Strategy:**

1 exp lymphatic filariasis/ (3271)
2 "lymphatic filarias?s".tw. (4091)
3 ("bancroftian filarias?s" or "brugia* filariasis" or "Malay* filarias?s" or elephantiasis or Elephantiases or microfilar?emia or hydrocele or "filarial lymphoedema" or "filarial lymphedema" or "filarial lymphooedema").tw. (6542)
4 adenolymphangitis.tw. (102)
5 (acute adj2 lymphangitis).tw. (58)
6 dermatolymphangioadenitis.tw. (52)
7 "B. pahangi".tw. (346)
8 "circulating filarial antigen".tw. (233)
9 brugiasis.tw. (1)

10 Wuchereria bancrofti/ (3149)
11 "Wuchereria bancrofti".tw. (2163)
12 exp Brugia/ (3243)
13 Brugia.tw. (2906)
14 "B. timori".tw. (77)
15 "B. malayi".tw. (975)
16 "W. bancrofti".tw. (817)
17 "ADL attacks".tw. (14)
18 ADLA.tw. (62)
19 dermato-lymphangio-adenitis.tw. (19)
20 1 or 2 or 3 or 4 or 5 or 6 or 7 or 8 or 9 or 10 or 11 or 12 or 13 or 14 or 15 or 16 or 17 or 18 or 19 (13871)
21 20 (13871)
22 limit 21 to yr="1990 -Current" (11535)

**Database: Global Health <1973 to 2023 Week 18>**

**Search Strategy:**
1 "lymphatic filarias?s".tw. (3319)
2 ("bancroftian filarias?s" or "brugia* filariasis" or "Malay* filarias?s" or elephantiasis or elephantiases or microfilar?emia or hydrocele or "filarial lymphoedema" or "filarial lymphedema" or "filarial lymphooedema").tw. (4006)
3 adenolymphangitis.tw. (81)
4 (acute adj2 lymphangitis).tw. (21)
5 dermatolymphangioadenitis.tw. (28)
6 "B. pahangi".tw. (608)
7 "circulating filarial antigen".tw. (164)
8 brugiasis.tw. (2)
9 "Wuchereria bancrofti".tw. (4268)
10 Brugia.tw. (3952)
11 "B. timori".tw. (113)
12 "B. malayi".tw. (1142)
13 "W. bancrofti".tw. (1008)
14 "ADL attacks".tw. (12)
15 ADLA.tw. (26)
16 dermato-lymphangio-adenitis.tw. (4)
17 lymphatic filariasis/ (2292)
18 Wuchereria bancrofti/ (4228)
19 exp Brugia/ (3832)
20 1 or 2 or 3 or 4 or 5 or 6 or 7 or 8 or 9 or 10 or 11 or 12 or 13 or 14 or 15 or 16 or 17 or 18 or 19 (9779)
21 limit 20 to yr="1990 -Current" (6578)

**Scopus**

TITLE-ABS-KEY ( "lymphatic filarias?s" OR "bancroftian filarias?s" OR "brugia* filariasis" OR "Malay* filarias?s" OR elephantiasis OR elephantiases OR microfilar?emia OR hydrocele OR "filarial lymphoedema" OR "filarial lymphedema" OR "filarial lymphooedema" OR adenolymphangitis OR ( acute W/2 lymphangitis ) OR dermatolymphangioadenitis OR "B. pahangi" OR "circulating filarial antigen" OR brugiasis OR "Wuchereria bancrofti" OR brugia OR "B. timori" OR "B. malayi" OR "W. bancrofti" OR "ADL attacks" OR adla OR dermato-lymphangio-adenitis ) AND ( LIMIT-TO ( PUBYEAR , 2022 ) OR LIMIT-TO ( PUBYEAR , 2021 ) OR LIMIT-TO ( PUBYEAR , 2020 ) OR LIMIT-TO ( PUBYEAR , 2019 ) OR LIMIT-TO ( PUBYEAR , 2018 ) OR LIMIT-TO ( PUBYEAR , 2017 ) OR LIMIT-TO ( PUBYEAR , 2016 ) OR LIMIT-TO ( PUBYEAR , 2015 ) OR LIMIT-TO ( PUBYEAR , 2014 ) OR LIMIT-TO ( PUBYEAR , 2013 ) OR LIMIT-TO ( PUBYEAR , 2012 ) OR LIMIT-TO ( PUBYEAR , 2011 ) OR LIMIT-TO ( PUBYEAR , 2010 ) OR LIMIT-TO ( PUBYEAR , 2009 ) OR LIMIT-TO ( PUBYEAR , 2008 ) OR LIMIT-TO ( PUBYEAR , 2007 ) OR LIMIT-TO ( PUBYEAR , 2006 ) OR LIMIT-TO ( PUBYEAR , 2005 ) OR LIMIT-TO ( PUBYEAR , 2004 ) OR LIMIT-TO ( PUBYEAR , 2003 ) OR LIMIT-TO ( PUBYEAR , 2002 ) OR LIMIT-TO ( PUBYEAR , 2001 ) OR LIMIT-TO ( PUBYEAR , 2000 ) OR LIMIT-TO ( PUBYEAR , 1999 ) OR LIMIT-TO ( PUBYEAR , 1998 ) OR LIMIT-TO ( PUBYEAR , 1997 ) OR LIMIT-TO ( PUBYEAR , 1996 ) OR LIMIT-TO ( PUBYEAR , 1995 ) OR LIMIT-TO ( PUBYEAR , 1994 ) OR LIMIT-TO ( PUBYEAR , 1993 ) OR LIMIT-TO ( PUBYEAR , 1992 ) OR LIMIT-TO ( PUBYEAR , 1991 ) OR LIMIT-TO ( PUBYEAR , 1990 ) )

**Web of Science Core Collection**

"lymphatic filarias?s" or "bancroftian filarias?s" or "brugia* filariasis" or "Malay* filarias?s" or elephantiasis or elephantiases or microfilar?emia or hydrocele or "filarial lymphoedema" or "filarial lymphedema" or "filarial lymphooedema" OR adenolymphangitis OR (acute near/2 lymphangitis) OR dermatolymphangioadenitis OR "B. pahangi" OR "circulating filarial antigen" OR brugiasis OR "Wuchereria bancrofti" OR Brugia OR "B. timori" OR "B. malayi" OR "W. bancrofti" OR "ADL attacks" OR ADLA OR dermato-lymphangio-adenitis (Topic) and 2022 or 2021 or 2020 or 2019 or 2018 or 2017 or 2016 or 2015 or 2014 or 2013 or 2012 or 2011 or 2010 or 2009 or 2008 or 2007 or 2006 or 2005 or 2004 or 2003 or 2002 or 2001 or 2000 or 1999 or 1998 or 1997 or 1996 or 1995 or 1994 or 1993 or 1992 or 1991 or 1990 (Publication Years)

**Cochrane Database of Systematic Reviews**

**Issue 5 of 12, May 2023**

**Cochrane Central Register of Controlled Trials**

**Issue 5 of 12, May 2023**

("lymphatic filarias?s" or "bancroftian filarias?s" or "brugia* filariasis" or "Malay* filarias?s" or elephantiasis or elephantiases or microfilar?emia or hydrocele or "filarial lymphoedema" or "filarial lymphedema" or "filarial lymphooedema" OR adenolymphangitis OR (acute near/2 lymphangitis) OR dermatolymphangioadenitis OR "B. pahangi" OR "circulating filarial antigen" OR brugiasis OR "Wuchereria bancrofti" OR Brugia OR "B. timori" OR "B. malayi" OR "W. bancrofti" OR "ADL attacks" OR ADLA OR dermato-lymphangio-adenitis):ti,ab,kw

Limited: 1990-Current

[**WHO Global Index Medicus**](https://pesquisa.bvsalud.org/gim/?u_filter%5B%5D=fulltext&u_filter%5B%5D=collection_gim&u_filter%5B%5D=mj_cluster&u_filter%5B%5D=type_of_study&u_filter%5B%5D=la&fb=&output=&lang=en&from=1&sort=&format=&count=&page=1&index=tw&q=%22lymphatic+filariasis%22+or+%22lymphatic+filariases%22+or+%22bancroftian+filariasis%22+or+%22bancroftian+filariases%22+or+%22brugia*+filariasis%22+or+%22Malay*+filarias%3Fs%22+or+%22Malay*+filariases%22+or+elephantiasis+or+elephantiases+or+microfilaremia+or+microfilaraemia+or+hydrocele+or+%22filarial+lymphoedema%22+or+%22filarial+lymphedema%22+or+%22filarial+lymphooedema%22+OR+adenolymphangitis+OR+%22acute+lymphangitis%22+OR+dermatolymphangioadenitis+OR++%22B.+pahangi%22+OR+%22circulating+filarial+antigen%22+OR+brugiasis+OR+%22Wuchereria+bancrofti%22+OR+Brugia+OR+%22B.+timori%22+OR+%22B.+malayi%22+OR+%22W.+bancrofti%22+OR+%22ADL+attacks%22+OR+ADLA+OR+dermato-lymphangio-adenitis+&where=&range_year_start=1990&range_year_end=2021&range_year_start=1990&range_year_end=2024)

Title, abstract, subject: "lymphatic filariasis" or "lymphatic filariases" or "bancroftian filariasis" or "bancroftian filariases" or "brugia* filariasis" or "Malay* filarias?s" or "Malay* filariases" or elephantiasis or elephantiases or microfilaremia or microfilaraemia or hydrocele or "filarial lymphoedema" or "filarial lymphedema" or "filarial lymphooedema" OR adenolymphangitis OR "acute lymphangitis" OR dermatolymphangioadenitis OR "B. pahangi" OR "circulating filarial antigen" OR brugiasis OR "Wuchereria bancrofti" OR Brugia OR "B. timori" OR "B. malayi" OR "W. bancrofti" OR "ADL attacks" OR ADLA OR dermato-lymphangio-adenitis

Limits: 1990-current

**Clinicaltrials.gov**

All : Lymphatic Filariases (searches Filariases: Elephantiasis, Wuchereria Bancrofti infection; Filariases: Elephantiasis, Wuchereria Bancrofti infection; Lymphatic).

**WHO ICTRP**

Phases are: All

Lymphatic filariasis, Lymphatic filariases

**African Journals Online**

Lymphatic filariasis

Elephantiasis

**Clinical Trials Registry India Ctri.nic.in**

Lymphatic filariasis
