# Supplementary material for: The lymphatic filariasis treatment study landscape: A systematic review of study characteristics and the case for an individual participant data platform
Source: PLoS Negl Trop Dis. 2024 Jan 16;18(1):e0011882. doi: 10.1371/journal.pntd.0011882 (PMC10817204; doi:10.1371/journal.pntd.0011882)
Supplement: S2 Text — (DOCX) [file pntd.0011882.s002.docx]

**References**

1. Dunyo SK, Nkrumah FK, Simonsen PE. Single-dose treatment of *Wuchereria bancrofti* infections with ivermectin and albendazole alone or in combination: evaluation of the potential for control at 12 months after treatment. Trans R Soc Trop Med Hyg. 2000; 94: 437-443. doi: 10.1016/s0035-9203(00)90135-4
2. Dunyo SK, Appawu M, Nkrumah FK, Baffoe-Wilmot A, Pedersen EM, Simonsen PE. Lymphatic filariasis on the coast of Ghana. Trans R Soc Trop Med Hyg. 1996; 90: 634-638. doi: 10.1016/s0035-9203(96)90414-9
3. Meyrowitsch DW, Simonsen PE, Magnussen P. Tolerance to diethylcarbamazine-medicated salt in individuals infected with *Onchocerca volvulus*. Trans R Soc Trop Med Hyg. 2000; 94: 444-448. doi: 10.1016/s0035-9203(00)90136-6
4. Shenoy RK, John A, Babu BS, Suma TK, Kumaraswami V. Two-year follow-up of the microfilaraemia of asymptomatic brugian filariasis, after treatment with two, annual, single doses of ivermectin, diethylcarbamazine and albendazole, in various combinations. Ann Trop Med Parasitol. 2000; 94: 607-614. doi: 10.1080/00034983.2000.11813583
5. Reddy GS, Vengatesvarlou N, Das PK, Vanamail P, Vijayan AP, Kala S, Pani SP. Tolerability and efficacy of single-dose diethyl carbamazine (DEC) or ivermectin in the clearance of *Wuchereria bancrofti* microfilaraemia in Pondicherry, south India. Trop Med Int Health. 2000; 5: 779-785. doi: 10.1046/j.1365-3156.2000.00644.x
6. Freedman DO, Plier DA, De Almeida AB, De Oliveira AL, Miranda J, Braga C. Effect of aggressive prolonged diethylcarbamazine therapy on circulating antigen levels in bancroftian filariasis. Trop Med Int Health. 2001; 6: 37-41. doi: 10.1046/j.1365-3156.2001.00666.x.
7. Freeman AR, Lammie PJ, Houston R, LaPointe MD, Streit TG, Jooste PL, Brissau JM, Lafontant JG, Addiss DG. A community-based trial for the control of lymphatic filariasis and iodine deficiency using salt fortified with diethylcarbamazine and iodine. Am J Trop Med Hyg. 2001; 65: 865-871. doi: 10.4269/ajtmh.2001.65.865
8. Hassan MM, Bahgat MA, Ali AE, Saleh A, El-Shafae OK, Abdel-Ghaffar MM, Mowafy NM. Circulating filarial antigens for monitoring the efficacy of ivermectin in treatment of filariasis. J Egypt Soc Parasitol. 2001; 31: 575-81.
9. Ismail MM, Jayakody RL, Weil GJ, Fernando D, De Silva MS, De Silva GA, Balasooriya WK. Long-term efficacy of single-dose combinations of albendazole, ivermectin and diethylcarbamazine for the treatment of bancroftian filariasis. Trans R Soc Trop Med Hyg. 2001; 95: 332-335. doi: 10.1016/s0035-9203(01)90257-3
10. Sahoo PK, Babu Geddam JJ, Satapathy AK, Mohanty MC, Das BK, Acharya AS, Mishra N, Ravindran B. Bancroftian filariasis: a 13-year follow-up study of asymptomatic microfilariae carriers and endemic normals in Orissa, India. Parasitology. 2002; 124: 191-201. doi: 10.1017/s0031182001001007
11. Beuria MK, Bal MS, Mandal NN, Das MK. Antigenemia at 10 years after diethylcarbamazine treatment of asymptomatic microfilaraemic individuals: marginal conversion to infection-free state. Parasite Immunol. 2002; 24: 109-111. doi: 10.1046/j.0141-9838.2001.00439.x
12. Bockarie MJ, Tisch DJ, Kastens W, Alexander ND, Dimber Z, Bockarie F, Ibam E, Alpers MP, Kazura JW. Mass treatment to eliminate filariasis in Papua New Guinea. N Engl J Med. 2002; 347: 1841-1848. doi: 10.1056/NEJMoa021309
13. Dunyo SK, Simonsen PE. Ivermectin and albendazole alone and in combination for the treatment of lymphatic filariasis in Ghana: follow-up after re-treatment with the combination. Trans R Soc Trop Med Hyg. 2002; 96: 189-192. doi: 10.1016/s0035-9203(02)90300-7
14. Pani S, Subramanyam Reddy G, Das L, Vanamail P, Hoti S, Ramesh J, Das P. Tolerability and efficacy of single dose albendazole, diethylcarbamazine citrate (DEC) or co-administration of albendazole with DEC in the clearance of *Wuchereria bancrofti* in asymptomatic microfilaraemic volunteers in Pondicherry, South India: a hospital-based study. Filaria J. 2002; 1: 1. doi: 10.1186/1475-2883-1-1
15. Ramzy RM, el-Setouhy M, Helmy H, Kandil AM, Ahmed ES, Farid HA, Faris R, Weil GJ. The impact of single-dose diethylcarbamazine treatment of bancroftian filariasis in a low-endemicity setting in Egypt. Am J Trop Med Hyg. 2002; 67: 196-200. doi: 10.4269/ajtmh.2002.67.196
16. Suma TK, Shenoy RK, Kumaraswami V. Efficacy and sustainability of a footcare programme in preventing acute attacks of adenolymphangitis in Brugian filariasis. Trop Med Int Health. 2002; 7: 763-766. doi: 10.1046/j.1365-3156.2002.00914.x
17. Weerasooriya MV, Gunawardena NK, Itoh M, Qiu XG, Kimura E. Prevalence and intensity of *Wuchereria bancrofti* antigenaemia in Sri Lanka by Og4C3 ELISA using filter paper-absorbed whole blood. Trans R Soc Trop Med Hyg. 2002; 96: 41-45. doi: 10.1016/s0035-9203(02)90234-8
18. Alli R, Bhunia B, Chhotray GP, Reddy MV, Harinath BC. Microscopic haematuria as an occult filarial infection in bhubaneshwar an endemic area for bancroftian filariasis. Indian J Clin Biochem. 2003; 18: 61-64. doi: 10.1007/BF02867666
19. Bernhard L, Bernhard P, Magnussen P. Management of Patients with Lymphoedema Caused by Filariasis in North-eastern Tanzania. Physiotherapy. 2003; 89: 743-749. doi: 10.1016/S0031-9406(05)60500-7
20. Chandra G, Rudra SK, Chatterjee SN. Treatment of microfilaraemics with DEC and its effect on vector infection and infectivity in tribal and non-tribal areas of Bankura district, West Bengal, India. J Commun Dis. 2003; 35: 36-9.
21. Chaubal NG, Pradhan GM, Chaubal JN, Ramani SK. Dance of live adult filarial worms is a reliable sign of scrotal filarial infection. J Ultrasound Med. 2003; 22: 765-769. doi: 10.7863/jum.2003.22.8.765
22. Das LK, Reddy GS, Pani SP. Some observations on the effect of Daflon (micronized purified flavonoid fraction of Rutaceae aurantiae) in bancroftian filarial lymphoedema. Filaria J. 2003; 2: 5. doi: 10.1186/1475-2883-2-5
23. Farid HA, Kamal SA, Weil GJ, Adham FK, Ramzy RM. Filariasis elimination in Egypt: impact of low microfilaraemics as sources of infection for mosquitoes. East Mediterr Health J. 2003; 9: 863-872.
24. Hoerauf A, Mand S, Fischer K, Kruppa T, Marfo-Debrekyei Y, Debrah AY, Pfarr KM, Adjei O, Büttner DW. Doxycycline as a novel strategy against bancroftian filariasis-depletion of *Wolbachia* endosymbionts from *Wuchereria bancrofti* and stop of microfilaria production. Med Microbiol Immunol. 2003; 192: 211-226. doi: 10.1007/s00430-002-0174-6

1. Keiser PB, Coulibaly YI, Keita F, Traore D, Diallo A, Diallo DA, Semnani RT, Doumbo OK, Traore SF, Klion AD, Nutman TB. Clinical characteristics of post-treatment reactions to ivermectin/albendazole for *Wuchereria bancrofti* in a region co-endemic for *Mansonella perstans.* Am J Trop Med Hyg. 2003; 69: 331-335.
2. Koyadun S, Bhumiratana A, Prikchu P. *Wuchereria bancrofti* antigenemia clearance among Myanmar migrants after biannual mass treatments with diethylcarbamazine, 300 mg oral-dose FILADEC tablet, in Southern Thailand. Southeast Asian J Trop Med Public Health. 2003; 34: 758-767
3. Makunde WH, Kamugisha LM, Massaga JJ, Makunde RW, Savael ZX, Akida J, Salum FM, Taylor MJ. Treatment of co-infection with bancroftian filariasis and onchocerciasis: a safety and efficacy study of albendazole with ivermectin compared to treatment of single infection with bancroftian filariasis. Filaria J. 2003; 2: 15. doi: 10.1186/1475-2883-2-15

1. Noroes J, Addiss D, Cedenho A, Figueredo-Silva J, Lima G, Dreyer G. Pathogenesis of filarial hydrocele: risk associated with intrascrotal nodules caused by death of adult *Wuchereria bancrofti*. Trans R Soc Trop Med Hyg. 2003; 97: 561-566. doi: 10.1016/s0035-9203(03)80029-9
2. Terhell AJ, Haarbrink M, van den Biggelaar A, Mangali A, Sartono E, Yazdanbakhsh M. Long-term follow-up of treatment with diethylcarbamazine on anti-filarial IgG4: dosage, compliance, and differential patterns in adults and children. Am J Trop Med Hyg. 2003; 68: 33-39.
3. Bhumiratana A, Siriaut C, Koyadun S, Satitvipawee P. Evaluation of a single oral dose of diethylcarbamazine 300 mg as provocative test and simultaneous treatment in Myanmar migrant workers with *Wuchereria bancrofti* infection in Thailand. Southeast Asian J Trop Med Public Health. 2004; 35: 591-598.
4. El Setouhy M, Ramzy RM, Ahmed ES, Kandil AM, Hussain O, Farid HA, Helmy H, Weil GJ. A randomized clinical trial comparing single- and multi-dose combination therapy with diethylcarbamazine and albendazole for treatment of bancroftian filariasis. Am J Trop Med Hyg. 2004; 70: 191-196.
5. Hussein O, El Setouhy M, Ahmed ES, Kandil AM, Ramzy RM, Helmy H, Weil GJ. Duplex Doppler sonographic assessment of the effects of diethylcarbamazine and albendazole therapy on adult filarial worms and adjacent host tissues in Bancroftian filariasis. Am J Trop Med Hyg. 2004; 71: 471-477.
6. Joseph A, Mony P, Prasad M, John S, Srikanth, Mathai D. The efficacies of affected-limb care with penicillin diethylcarbamazine, the combination of both drugs or antibiotic ointment, in the prevention of acute adenolymphangitis during bancroftian filariasis. Ann Trop Med Parasitol. 2004; 98: 685-696. doi: 10.1179/000349804225021451
7. Kshirsagar NA, Gogtay NJ, Garg BS, Deshmukh PR, Rajgor DD, Kadam VS, Kirodian BG, Ingole NS, Mehendale AM, Fleckenstein L, Karbwang J, Lazdins-Helds JK. Safety, tolerability, efficacy and plasma concentrations of diethylcarbamazine and albendazole co-administration in a field study in an area endemic for lymphatic filariasis in India. Trans R Soc Trop Med Hyg. 2004; 98: 205-217. doi: 10.1016/s0035-9203(03)00044-0
8. Meyrowitsch DW, Simonsen PE, Magesa SM. A 26-year follow-up of bancroftian filariasis in two communities in north-eastern Tanzania. Ann Trop Med Parasitol. 2004; 98: 155-169. doi: 10.1179/000349804225003172
9. Subramanyam GR, Vengatesvarlou N, Das PK, Vanamail P, Vijayan AP, Kala S, Pani SP. Tolerability and efficacy of single-dose diethyl carbamazine (DEC) or ivermectin in the clearance of *Wuchereria bancrofti* microfilaraemia in Pondicherry, south India. Trop Med Int Health. 2000; 5: 779-785. doi: 10.1046/j.1365-3156.2000.00644.x
10. Simonsen PE, Magesa SM, Dunyo SK, Malecela-Lazaro MN, Michael E. The effect of single dose ivermectin alone or in combination with albendazole on *Wuchereria bancrofti* infection in primary school children in Tanzania. Trans R Soc Trop Med Hyg. 2004; 98: 462-472. doi: 10.1016/j.trstmh.2003.12.005
11. Simonsen PE, Meyrowitsch DW, Mukoko DA, Pedersen EM, Malecela-Lazaro MN, Rwegoshora RT, Ouma JH, Masese N, Jaoko WG, Michael E. The effect of repeated half-yearly diethylcarbamazine mass treatment on *Wuchereria bancrofti* infection and transmission in two East African communities with different levels of endemicity. Am J Trop Med Hyg. 2004 ; 70: 63-71.
12. Washington CH, Radday J, Streit TG, Boyd HA, Beach MJ, Addiss DG, Lovince R, Lovegrove MC, Lafontant JG, Lammie PJ, Hightower AW. Spatial clustering of filarial transmission before and after a Mass Drug Administration in a setting of low infection prevalence. Filaria J. 2004; 3:3. doi: 10.1186/1475-2883-3-3.
13. Bhumiratana A, Koyadun S, Srisuphanunt M, Satitvipawee P, Limpairojn N, Gaewchaiyo G. Border and imported bancroftian filariases: baseline seroprevalence in sentinel populations exposed to infections with *Wuchereria bancrofti* and concomitant HIV at the start of diethylcarbamazine mass treatment in Thailand. Southeast Asian J Trop Med Public Health. 2005; 36: 390-407.
14. Farid HA, Hammad RE, Hassan MM, Ramzy RM, El Setouhy M, Weil GJ. Effects of combined diethylcarbamazine and albendazole treatment of bancroftian filariasis on parasite uptake and development in *Culex pipiens L*. Am J Trop Med Hyg. 2005; 73: 108-114.
15. Fox LM, Furness BW, Haser JK, Desire D, Brissau JM, Milord MD, Lafontant J, Lammie PJ, Beach MJ. Tolerance and efficacy of combined diethylcarbamazine and albendazole for treatment of *Wuchereria bancrofti* and intestinal helminth infections in Haitian children. Am J Trop Med Hyg. 2005; 73: 115-121.
16. Moss DM, Priest JW, Boyd A, Weinkopff T, Kucerova Z, Beach MJ, Lammie PJ. Multiplex bead assay for serum samples from children in Haiti enrolled in a drug study for the treatment of lymphatic filariasis. Am J Trop Med Hyg. 2011; 85: 229-237. doi: 10.4269/ajtmh.2011.11-0029
17. Kerketta AS, Babu BV, Rath K, Jangid PK, Nayak AN, Kar SK. A randomized clinical trial to compare the efficacy of three treatment regimens along with footcare in the morbidity management of filarial lymphoedema. Trop Med Int Health. 2005; 10: 698-705. doi: 10.1111/j.1365-3156.2005.01442.x
18. Oqueka T, Supali T, Ismid IS, Purnomo, Rückert P, Bradley M, Fischer P. Impact of two rounds of mass drug administration using diethylcarbamazine combined with albendazole on the prevalence of *Brugia timori* and of intestinal helminths on Alor Island, Indonesia. Filaria J. 2005; 4: 5. doi: 10.1186/1475-2883-4-5
19. Sahoo PK, Satapathy AK, Michael E, Ravindran B. Concomitant parasitism: bancroftian filariasis and intestinal helminths and response to albendazole. Am J Trop Med Hyg. 2005; 73: 877-880.
20. Simonsen PE, Magesa SM, Meyrowitsch DW, Malecela-Lazaro MN, Rwegoshora RT, Jaoko WG, Michael E. The effect of eight half-yearly single-dose treatments with DEC on *Wuchereria bancrofti* circulating antigenaemia. Trans R Soc Trop Med Hyg. 2005; 99: 541-547. doi: 10.1016/j.trstmh.2004.11.016.
21. Siriaut C, Bhumiratana A, Koyadun S, Anurat K, Satitvipawee P. Short-term effects of treatment with 300 mg oral-dose diethylcarbamazine on nocturnally periodic *Wuchereria bancrofti* microfilaremia and antigenemia. Southeast Asian J Trop Med Public Health. 2005; 36: 832-840.
22. Stolk WA, VAN Oortmarssen GJ, Pani SP, DE Vlas SJ, Subramanian S, DAS PK, Habbema JD. Effects of ivermectin and diethylcarbamazine on microfilariae and overall microfilaria production in bancroftian filariasis. Am J Trop Med Hyg. 2005; 73: 881-887.
23. Taylor MJ, Makunde WH, McGarry HF, Turner JD, Mand S, Hoerauf A. Macrofilaricidal activity after doxycycline treatment of *Wuchereria bancrofti*: a double-blind, randomised placebo-controlled trial. Lancet. 2005; 365: 2116-2121. doi: 10.1016/S0140-6736(05)66591-9
24. Helmy H, Weil GJ, Ellethy AS, Ahmed ES, Setouhy ME, Ramzy RM. Bancroftian filariasis: effect of repeated treatment with diethylcarbamazine and albendazole on microfilaraemia, antigenaemia and antifilarial antibodies. Trans R Soc Trop Med Hyg. 2006; 100: 656-662. doi: 10.1016/j.trstmh.2005.08.015
25. Debrah AY, Mand S, Specht S, Marfo-Debrekyei Y, Batsa L, Pfarr K, Larbi J, Lawson B, Taylor M, Adjei O, Hoerauf A. Doxycycline reduces plasma VEGF-C/sVEGFR-3 and improves pathology in lymphatic filariasis. PLoS Pathog. 2006; 2: e92. doi: 10.1371/journal.ppat.0020092
26. Dreyer G, Addiss D, Williamson J, Norões J. Efficacy of co-administered diethylcarbamazine and albendazole against adult *Wuchereria bancrofti*. Trans R Soc Trop Med Hyg. 2006; 100: 1118-1125. doi: 10.1016/j.trstmh.2006.04.006
27. Makunde WH, Kamugisha LM, Makunde RA, Malecela MN, Kitua AY. Hospital-based safety and tolerability study to assess efficacy of oral doxycycline in the treatment of *Wuchereria bancrofti* infection in north-eastern Tanzania. Tanzan Health Res Bull. 2006; 8:128-133. doi: 10.4314/thrb.v8i3.45109
28. Sunish IP, Rajendran R, Mani TR, Munirathinam A, Reuben R, Dash AP. Impact of single dose of diethylcarbamazine and other antifilarial drug combinations on bancroftian filarial infection variables: assessment after 2 years. Parasitol Int. 2006; 55: 233-236. doi: 10.1016/j.parint.2006.05.003
29. Turner JD, Mand S, Debrah AY, Muehlfeld J, Pfarr K, McGarry HF, Adjei O, Taylor MJ, Hoerauf A. A randomized, double-blind clinical trial of a 3-week course of doxycycline plus albendazole and ivermectin for the treatment of *Wuchereria bancrofti* infection. Clin Infect Dis. 2006; 42: 1081-1089. doi: 10.1086/501351
30. Yongyuth P, Koyadun S, Jaturabundit N, Sampuch A, Bhumiratana A. Efficacy of a single-dose treatment with 300 mg diethylcarbamazine and a combination of 400 mg albendazole in reduction of *Wuchereria bancrofti* antigenemia and concomitant geohelminths in Myanmar migrants in Southern Thailand. J Med Assoc Thai. 2006; 89: 1237-1248.
31. Bockarie MJ, Tavul L, Ibam I, Kastens W, Hazlett F, Tisch DJ, Alpers MP, Kazura JW. Efficacy of single-dose diethylcarbamazine compared with diethylcarbamazine combined with albendazole against *Wuchereria bancrofti* infection in Papua New Guinea. Am J Trop Med Hyg. 2007; 76: 62-6.
32. Chandra G, Chatterjee SN, Das S, Sarkar N. Lymphatic filariasis in the coastal areas of Digha, West Bengal, India. Trop Doct. 2007; 37: 136-139. doi: 10.1258/004947507781524737
33. Dixit V, Gupta AK, Bisen PS, Prasad GB, Harinath BC. Serum immune complexes as diagnostic and therapeutic markers in lymphatic filariasis. J Clin Lab Anal. 2007; 21: 114-118. doi: 10.1002/jcla.20116
34. Malla N, Elango A, Pani SP, Mahajan RC. Kinetics of microfilaraemia & antigenaemia status by Og(4)C(3) ELISA in bancroftian filariasis. Indian J Med Res. 2007; 126: 567-574.
35. Nielsen NO, Simonsen PE, Dalgaard P, Krarup H, Magnussen P, Magesa S, Friis H. Effect of diethylcarbamazine on HIV load, CD4%, and CD4/CD8 ratio in HIV-infected adult Tanzanians with or without lymphatic filariasis: randomized double-blind and placebo-controlled cross-over trial. Am J Trop Med Hyg. 2007; 77: 507-513.
36. Ramaiah KD, Vanamail P, Das PK. Changes in *Wuchereria bancrofti* infection in a highly endemic community following 10 rounds of mass administration of diethylcarbamazine. Trans R Soc Trop Med Hyg. 2007; 101: 250-255. doi: 10.1016/j.trstmh.2006.05.007
37. Rizzo JA, Belo C, Lins R, Dreyer G. Children and adolescents infected with *Wuchereria bancrofti* in Greater Recife, Brazil: a randomized, year-long clinical trial of single treatments with diethylcarbamazine or diethylcarbamazine-albendazole. Ann Trop Med Parasitol. 2007; 101: 423-433. doi: 10.1179/136485907X176517
38. Wijesinghe RS, Wickremasinghe AR, Ekanayake S, Perera MS. Efficacy of a limb-care regime in preventing acute adenolymphangitis in patients with lymphoedema caused by bancroftian filariasis, in Colombo, Sri Lanka. Ann Trop Med Parasitol. 2007; 101: 487-497. doi: 10.1179/136485907X193806
39. Chandra G, Paramanik M. Effect of single to triple dose DEC on microfilaremics up to 5 years. Parasitol Res. 2008; 103: 1279-1282. doi: 10.1007/s00436-008-1126-x
40. Supali T, Djuardi Y, Pfarr KM, Wibowo H, Taylor MJ, Hoerauf A, Houwing-Duistermaat JJ, Yazdanbakhsh M, Sartono E. Doxycycline treatment of *Brugia malayi*-infected persons reduces microfilaremia and adverse reactions after diethylcarbamazine and albendazole treatment. Clin Infect Dis. 2008; 46: 1385-1393. doi: 10.1086/586753
41. Weil GJ, Kastens W, Susapu M, Laney SJ, Williams SA, King CL, Kazura JW, Bockarie MJ. The impact of repeated rounds of mass drug administration with diethylcarbamazine plus albendazole on bancroftian filariasis in Papua New Guinea. PLoS Negl Trop Dis. 2008; 2: e344. doi: 10.1371/journal.pntd.0000344
42. Coulibaly YI, Dembele B, Diallo AA, Lipner EM, Doumbia SS, Coulibaly SY, Konate S, Diallo DA, Yalcouye D, Kubofcik J, Doumbo OK, Traore AK, Keita AD, Fay MP, Traore SF, Nutman TB, Klion AD. A randomized trial of doxycycline for *Mansonella perstans* infection. N Engl J Med. 2009; 361: 1448-1458. doi: 10.1056/NEJMoa0900863
43. Debrah AY, Mand S, Marfo-Debrekyei Y, Batsa L, Pfarr K, Lawson B, Taylor M, Adjei O, Hoerauf A. Reduction in levels of plasma vascular endothelial growth factor-A and improvement in hydrocele patients by targeting endosymbiotic Wolbachia sp. in *Wuchereria bancrofti* with doxycycline. Am J Trop Med Hyg. 2009; 80: 956-963.
44. El-Shazly AM, Saker TI, El-Fayoumy KN, Aboulmagd AA, El-Ghareeb AS, Zalouk TK, Abdel-Tawab AH. The kinetics of microfilaraemia and antigenaemia status among asymptomatic bancroftian filariasis before and after treatment. J Egypt Soc Parasitol. 2009; 39: 191-204.
45. Mand S, Pfarr K, Sahoo PK, Satapathy AK, Specht S, Klarmann U, Debrah AY, Ravindran B, Hoerauf A. Macrofilaricidal activity and amelioration of lymphatic pathology in bancroftian filariasis after 3 weeks of doxycycline followed by single-dose diethylcarbamazine. Am J Trop Med Hyg. 2009; 81: 702-711. doi: 10.4269/ajtmh.2009.09-0155
46. Nielsen NO, Simonsen PE, Kaestel P, Krarup H, Magnussen P, Magesa S, Friis H. Micronutrient status indicators in individuals single- or double-infected with HIV and *Wuchereria bancrofti* before and after DEC treatment. Trop Med Int Health. 2009; 14: 44-53. doi: 10.1111/j.1365-3156.2008.02180.x
47. Petersen HH, Nielsen NO, Monrad J, Magesa SM, Simonsen PE. The effect of HIV on filarial-specific antibody response before and after treatment with diethylcarbamazine in *Wuchereria bancrofti* infected individuals. Parasitol Int. 2009; 58: 141-144. doi: 10.1016/j.parint.2009.01.003
48. Shenoy RK, Suma TK, Kumaraswami V, Rahmah N, Dhananjayan G, Padma S. Antifilarial drugs, in the doses employed in mass drug administrations by the Global Programme to Eliminate Lymphatic Filariasis, reverse lymphatic pathology in children with *Brugia malayi* infection. Ann Trop Med Parasitol. 2009; 103: 235-247. doi: 10.1179/136485909X398249
49. Thomas G, Richards FO Jr, Eigege A, Dakum NK, Azzuwut MP, Sarki J, Gontor I, Abimiku J, Ogah G, Jindau MY, Jiya JY, Miri ES. A pilot program of mass surgery weeks for treatment of hydrocele due to lymphatic filariasis in central Nigeria. Am J Trop Med Hyg. 2009; 80: 447-451.
50. Addiss DG, Louis-Charles J, Roberts J, Leconte F, Wendt JM, Milord MD, Lammie PJ, Dreyer G. Feasibility and effectiveness of basic lymphedema management in Leogane, Haiti, an area endemic for bancroftian filariasis. PLoS Negl Trop Dis. 2010; 4: e668. doi: 10.1371/journal.pntd.0000668
51. Eddy BA, Blackstock AJ, Williamson JM, Addiss DG, Streit TG, Beau de Rochars VM, Fox LM. A longitudinal analysis of the effect of mass drug administration on acute inflammatory episodes and disease progression in lymphedema patients in Leogane, Haiti. Am J Trop Med Hyg. 2014; 90: 80-88. doi: 10.4269/ajtmh.13-0317
52. Addiss DG, Michel MC, Michelus A, Radday J, Billhimer W, Louis-Charles J, Roberts JM, Kramp K, Dahl BA, Keswick B. Evaluation of antibacterial soap in the management of lymphoedema in Leogane, Haiti. Trans R Soc Trop Med Hyg. 2011; 105: 58-60. doi: 10.1016/j.trstmh.2010.08.011.
53. Bhumiratana A, Pechgit P, Koyadun S, Siriaut C, Yongyuth P. Imported bancroftian filariasis: diethylcarbamazine response and benzimidazole susceptibility of *Wuchereria bancrofti* in dynamic cross-border migrant population targeted by the National Program to Eliminate Lymphatic Filariasis in South Thailand. Acta Trop. 2010; 113: 121-128. doi: 10.1016/j.actatropica.2009.10.004
54. Dembele B, Coulibaly YI, Dolo H, Konate S, Coulibaly SY, Sanogo D, Soumaoro L, Coulibaly ME, Doumbia SS, Diallo AA, Traore SF, Diaman Keita A, Fay MP, Nutman TB, Klion AD. Use of high-dose, twice-yearly albendazole and ivermectin to suppress *Wuchereria bancrofti* microfilarial levels. Clin Infect Dis. 2010; 51: 1229-1235. doi: 10.1086/657063
55. Dixit V, Baghel P, Gupta A, Bisen P, Prasad GBKS. Interruption of Annual Single Dose DEC Regimen Administration Fails to Affect Transmission Intensity Even in a Situation of Filarial Low Endemicity. Int Medical J. 2010; 17: 117-121.
56. Hoti SL, Pani SP, Vanamail P, Athisaya Mary K, Das LK, Das PK. Effect of a single dose of diethylcarbamazine, albendazole or both on the clearance of *Wuchereria bancrofti* microfilariae and antigenaemia among microfilaria carriers: a randomized trial. Natl Med J India. 2010; 23: 72-76.
57. Norões J, Dreyer G. A mechanism for chronic filarial hydrocele with implications for its surgical repair. PLoS Negl Trop Dis. 2010; 4: e695. doi: 10.1371/journal.pntd.0000695.
58. Sanprasert V, Sujariyakul A, Nuchprayoon S. A single dose of doxycycline in combination with diethylcarbamazine for treatment of bancroftian filariasis. Southeast Asian J Trop Med Public Health. 2010; 41: 800-812.
59. Simonsen PE, Pedersen EM, Rwegoshora RT, Malecela MN, Derua YA, Magesa SM. Lymphatic filariasis control in Tanzania: effect of repeated mass drug administration with ivermectin and albendazole on infection and transmission. PLoS Negl Trop Dis. 2010; 4: e696. doi: 10.1371/journal.pntd.0000696
60. Wamae CN, Njenga SM, Ngugi BM, Mbui J, Njaanake HK. Evaluation of effectiveness of diethylcarbamazine/albendazole combination in reduction of *Wuchereria bancrofti* infection using multiple infection parameters. Acta Trop. 2011; 120: S33-8. doi: 10.1016/j.actatropica.2010.09.009
61. Bose K, Aggithaya G. An integrative treatment for lower limb lymphoedema in India. Br J Community Nurs. 2011; 16: S22-S27. doi: 10.12968/bjcn.2011.16.Sup10.S22.
62. Debrah AY, Mand S, Marfo-Debrekyei Y, Batsa L, Albers A, Specht S, Klarmann U, Pfarr K, Adjei O, Hoerauf A. Macrofilaricidal Activity in *Wuchereria bancrofti* after 2 Weeks Treatment with a Combination of Rifampicin plus Doxycycline. J Parasitol Res. 2011; 2011: 201617. doi: 10.1155/2011/201617
63. El-Nahas H, El-Shazly A, Abulhassan M, Nabih N, Mousa N. Impact of basic lymphedema management and antifilarial treatment on acute dermatolymphangioadenitis episodes and filarial antigenaemia. J Glob Infect Dis. 2011; 3: 227-232. doi: 10.4103/0974-777X.83527.
64. Jullien P, Somé Jd, Brantus P, Bougma RW, Bamba I, Kyelem D. Efficacy of home-based lymphoedema management in reducing acute attacks in subjects with lymphatic filariasis in Burkina Faso. Acta Trop. 2011; 120: S55-S61. doi: 10.1016/j.actatropica.2011.03.007.
65. Njenga SM, Mwandawiro CS, Wamae CN, Mukoko DA, Omar AA, Shimada M, Bockarie MJ, Molyneux DH. Sustained reduction in prevalence of lymphatic filariasis infection in spite of missed rounds of mass drug administration in an area under mosquito nets for malaria control. Parasit Vectors. 2011; 4: 90. doi: 10.1186/1756-3305-4-90.
66. Njenga SM, Wamae CN, Njomo DW, Mwandawiro CS, Molyneux DH: Impact of two rounds of mass treatment with diethylcarbamazine plus albendazole on *Wuchereria bancrofti* infection and the sensitivity of immunochromatographic test in Malindi, Kenya. Trans R Soc Trop Med

Hyg 2008; 102: 1017-1024

1. Singh V, Sinha RJ, Sankhwar SN, Kumar V. Reconstructive surgery for penoscrotal filarial lymphedema: a decade of experience and follow-up. Urology. 2011; 77: 1228-1231. doi: 10.1016/j.urology.2010.10.026
2. Mand S, Debrah AY, Klarmann U, Batsa L, Marfo-Debrekyei Y, Kwarteng A, Specht S, Belda-Domene A, Fimmers R, Taylor M, Adjei O, Hoerauf A. Doxycycline improves filarial lymphedema independent of active filarial infection: a randomized controlled trial. Clin Infect Dis. 2012; 55: 621-630. doi: 10.1093/cid/cis486
3. Steel C, Kubofcik J, Ottesen EA, Nutman TB. Antibody to the filarial antigen Wb123 reflects reduced transmission and decreased exposure in children born following single mass drug administration (MDA). PLoS Negl Trop Dis. 2012; 6: e1940. doi: 10.1371/journal.pntd.0001940
4. Budge PJ, Little KM, Mues KE, Kennedy ED, Prakash A, Rout J, Fox LM. Impact of community-based lymphedema management on perceived disability among patients with lymphatic filariasis in Orissa State, India. PLoS Negl Trop Dis. 2013; 7: e2100. doi: 10.1371/journal.pntd.0002100
5. Gayen P, Nayak A, Saini P, Mukherjee N, Maitra S, Sarkar P, Sinha Babu SP. A double-blind controlled field trial of doxycycline and albendazole in combination for the treatment of bancroftian filariasis in India. Acta Trop. 2013; 125: 150-156. doi: 10.1016/j.actatropica.2012.10.011
6. Narahari SR, Bose KS, Aggithaya MG, Swamy GK, Ryan TJ, Unnikrishnan B, Washington RG, Rao BP, Rajagopala S, Manjula K, Vandana U, Sreemol TA, Rojith M, Salimani SY, Shefuvan M. Community level morbidity control of lymphoedema using self care and integrative treatment in two lymphatic filariasis endemic districts of South India: a non randomized interventional study. Trans R Soc Trop Med Hyg. 2013; 107: 566-77. doi: 10.1093/trstmh/trt054
7. Prasad GP, Swamy G, & Naidu ML. A study on Kandughna taila in filarial lymphangitis and ulcers. Int J Ayurveda Pharma Res. 2013; 1: 31-37.
8. Sankari T, Hoti SL, Das LK, Govindaraj V, Das PK. Effect of Diethylcarbamazine (DEC) on prostaglandin levels in *Wuchereria bancrofti* infected microfilaraemics. Parasitol Res. 2013; 112: 2353-2359. doi: 10.1007/s00436-013-3399-y
9. Shenoy RK, Suma TK, & Kumaraswami V, Noordin R, Dhananjayan G, Padma, S. Anti-filarial drugs in doses employed in Mass Drug Administration in lymphatic filariasis elimination programme provide sustained benefits to children with *Brugia malayi* infection: A long-term follow up study. J Commun Dis. 2013; 45: 123-134.
10. Shenoy RK, Suma TK, Kumaraswami V, Rahmah N, Dhananjayan G, Padma S, Abhilash G, Ramesh C. Preliminary findings from a cross sectional study on lymphatic filariasis in children, in an area of India endemic for *Brugia malayi* infection. Ann Trop Med Parasitol. 2007; 101: 205-213. doi: 10.1179/136485907X154548
11. Shenoy RK, Suma TK, Kumaraswami V, Rahmah N, Dhananjayan G, Padma S. Antifilarial drugs, in the doses employed in mass drug administrations by the Global Programme to Eliminate Lymphatic Filariasis, reverse lymphatic pathology in children with *Brugia malayi* infection. Ann Trop Med Parasitol. 2009; 103: 235-247. doi: 10.1179/136485909X398249
12. Mues KE, Deming M, Kleinbaum DG, Budge PJ, Klein M, Leon JS, Prakash A, Rout J, Fox LM. Impact of a community-based lymphedema management program on episodes of Adenolymphangitis (ADLA) and lymphedema progression--Odisha State, India. PLoS Negl Trop Dis. 2014; 8: e3140. doi: 10.1371/journal.pntd.0003140
13. Adhikari P, Haldar S, Ghosh NR, Mandal MM, Haldar JP. Prevalence of Bancroftian filariasis in Burdwan District, west Bengal: a comparative study between colliery and non-colliery areas. J Commun Dis. 1994; 26: 6-13.
14. Shriram AN, Krishnamoorthy K, Sivan A, Saha BP, Kumaraswami V, Vijayachari P. Impact of MDA and the prospects of elimination of the lone focus of diurnally sub periodic lymphatic filariasis in Nicobar Islands, India. Acta Trop. 2014; 133: 93-7. doi: 10.1016/j.actatropica.2014.02.004.
15. Aggithaya MG, Narahari SR, Ryan TJ. Yoga for correction of lymphedema's impairment of gait as an adjunct to lymphatic drainage: A pilot observational study. Int J Yoga. 2015; 8: 54-61. doi: 10.4103/0973-6131.146063
16. De Britto RL, Vanamail P, Sankari T, Vijayalakshmi G, Das LK, Pani SP. Enhanced efficacy of sequential administration of Albendazole for the clearance of *Wuchereria bancrofti* infection: Double blind RCT. Trop Biomed. 2015; 32: 198-209.
17. Kar SK, Dwibedi B, Kerketa AS, Maharana A, Panda SS, Mohanty PC, Horton J, Ramachandran CP. A randomized controlled trial of increased dose and frequency of albendazole with standard dose DEC for treatment of *Wuchereria bancrofti* microfilaremics in Odisha, India. PLoS Negl Trop Dis. 2015; 9: e0003583. doi: 10.1371/journal.pntd.0003583
18. Pion SD, Chesnais CB, Bopda J, Louya F, Fischer PU, Majewski AC, Weil GJ, Boussinesq M, Missamou F. The impact of two semiannual treatments with albendazole alone on lymphatic filariasis and soil-transmitted helminth infections: a community-based study in the Republic of Congo. Am J Trop Med Hyg. 2015; 92: 959-966. doi: 10.4269/ajtmh.14-0661

1. Tafatatha TT, Ngwira BM, Taegtmeyer M, Phiri AJ, Wilson TP, Banda LG, Piston WN, Koole O, Horton J, French N. Randomised controlled clinical trial of increased dose and frequency of albendazole and ivermectin on *Wuchereria bancrofti* microfilarial clearance in northern Malawi. Trans R Soc Trop Med Hyg. 2015; 109: 393-399. doi: 10.1093/trstmh/trv027
2. Talaat KR, Babu S, Menon P, Kumarasamy N, Sharma J, Arumugam J, Dhakshinamurthy K, Srinivasan R, Poongulali S, Gu W, Fay MP, Swaminathan S, Nutman TB. Treatment of W. bancrofti (Wb) in HIV/Wb coinfections in South India. PLoS Negl Trop Dis. 2015; 9: e0003622. doi: 10.1371/journal.pntd.0003622
3. Soares H, Rocha A, Aguiar-Santos A, da Silva Santos, B, Melo C, Andrade A. Terapia complexa descongestiva com uso de material alternativo na redução e controle do linfedema em pacientes de área endêmica de filariose: um ensaio clínico. Fisioterapia e Pesquisa. 2016; 23: 268-277. doi: 10.1590/1809-2950/15476523032016
4. Kroidl I, Saathof E, Maganga L, Clowes P, Maboko L, Hoerauf A, Makunde WH, Haule A, Mviombo P, Pitter B, Mgeni N, Mabuye J, Kowuor D, Mwingira U, Malecela MN, Löscher T, Hoelscher M. Prevalence of Lymphatic Filariasis and Treatment Effectiveness of Albendazole/ Ivermectin in Individuals with HIV Co-infection in Southwest-Tanzania. PLoS Negl Trop Dis. 2016; 10: e0004618. doi: 10.1371/journal.pntd.0004618
5. Mnkai J, Marandu TF, Mhidze J, Urio A, Maganga L, Haule A, Kavishe G, Ntapara E, Chiwerengo N, Clowes P, Horn S, Mosoba M, Lazarus W, Ngenya A, Kalinga A, Debrah A, Rieß F, Saathoff E, Geldmacher C, Hoerauf A, Hoelscher M, Chachage M, Kroidl I. Step towards elimination of *Wuchereria bancrofti* in Southwest Tanzania 10 years after mass drug administration with Albendazole and Ivermectin. PLoS Negl Trop Dis. 2022; 16: e0010044. doi: 10.1371/journal.pntd.0010044
6. Thomsen EK, Sanuku N, Baea M, Satofan S, Maki E, Lombore B, Schmidt MS, Siba PM, Weil GJ, Kazura JW, Fleckenstein LL, King CL. Efficacy, Safety, and Pharmacokinetics of Coadministered Diethylcarbamazine, Albendazole, and Ivermectin for Treatment of Bancroftian Filariasis. Clin Infect Dis. 2016; 62: 334-341. doi: 10.1093/cid/civ882
7. de Souza DK, Owusu IO, Otchere J, Adimazoya M, Frempong K, Ahorlu CS, Boakye DA, Wilson MD. An evaluation of Wb123 antibody ELISA in individuals treated with ivermectin and albendazole, and implementation challenges in Africa. Pan Afr Med J. 2017; 27: 65. doi: 10.11604/pamj.2017.27.65.11004
8. Kanamitie JN, Ahorlu CS, Otchere J, Aboagye-Antwi F, Kwansa-Bentum B, Boakye DA, Biritwum NK, Wilson MD, de Souza DK. Twelve-month longitudinal parasitological assessment of lymphatic filariasis-positive individuals: impact of a biannual treatment with ivermectin and albendazole. Trop Med Int Health. 2017; 22: 1451-1456. doi: 10.1111/tmi.12974
9. Kar SK, Dwibedi B, Das BK, Agrawala BK, Ramachandran CP, Horton J. Lymphatic pathology in asymptomatic and symptomatic children with *Wuchereria bancrofti* infection in children from Odisha, India and its reversal with DEC and albendazole treatment. PLoS Negl Trop Dis. 2017; 11: e0005631. doi: 10.1371/journal.pntd.0005631
10. Kshirsagar NA, Gogtay NJ, Garg BS, Deshmukh PR, Rajgor DD, Kadam VS, Thakur PA, Gupta A, Ingole NS, Lazdins-Helds JK. Efficacy and tolerability of treatment with single doses of diethylcarbamazine (DEC) and DEC plus albendazole (ABZ) for three consecutive years in lymphatic filariasis: a field study in India. Parasitol Res. 2017; 116: 2683-2694. doi: 10.1007/s00436-017-5577-9
11. Yahathugoda TC, Weerasooriya MV, Samarawickrema WA, Kimura E, Itoh M. Impact of two follow-up schemes on morbidity management and disability prevention (MMDP) programme for filarial lymphedema in Matara, Sri Lanka. Parasitol Int. 2018; 67: 176-183. doi: 10.1016/j.parint.2017.11.005
12. da Silva JSF, Braga C, Duarte FM, Oliveira P, Feitosa Luna C, Marcondes M, Araújo J, Grilis MR, de Souza Melo PFA, Brandão E, Rocha A. Effectiveness of annual single doses of diethylcarbamazine citrate among bancroftian filariasis infected individuals in an endemic area under mass drug administration in Brazil. Pathog Glob Health. 2018; 112: 274-280. doi: 10.1080/20477724.2018.1498821
13. King CL, Suamani J, Sanuku N, Cheng YC, Satofan S, Mancuso B, Goss CW, Robinson LJ, Siba PM, Weil GJ, Kazura JW. A Trial of a Triple-Drug Treatment for Lymphatic Filariasis. N Engl J Med. 2018; 379: 1801-1810. doi: 10.1056/NEJMoa1706854
14. King CL, Weil GJ, Kazura JW. Single-Dose Triple-Drug Therapy for *Wuchereria bancrofti* - 5-Year Follow-up. N Engl J Med. 2020; 382: 1956-1957. doi: 10.1056/NEJMc1914262
15. Sulianti A, Agoes R, Hilmanto D, Adikara, T. Bio-psychology review on Low Level Laser Therapy management to overcome the condition of elephantiasis in chronic filariasis. IOP Conference Series: Materials Science and Engineering. 2018; 434: 012130. doi: 10.1088/1757-899X/434/1/012130
16. Chilgar RM, Khade S, Chen HC, Ciudad P, Yeo MS, Kiranantawat K, Maruccia M, Li K, Zhang YX, Nicoli F. Surgical Treatment of Advanced Lymphatic Filariasis of Lower Extremity Combining Vascularized Lymph Node Transfer and Excisional Procedures. Lymphat Res Biol. 2019; 17: 637-646. doi: 10.1089/lrb.2018.0058
17. Edi C, Bjerum CM, Ouattara AF, Chhonker YS, Penali LK, Méité A, Koudou BG, Weil GJ, King CL, Murry DJ. Pharmacokinetics, safety, and efficacy of a single co-administered dose of diethylcarbamazine, albendazole and ivermectin in adults with and without *Wuchereria bancrofti* infection in Côte d'Ivoire. PLoS Negl Trop Dis. 2019; 13: e0007325. doi: 10.1371/journal.pntd.0007325
18. Lunge VR. Prevalence of lymphatic filariasis in a tribal area of Maharashtra. Int J Community Med Public Health. 2019; 6: 533–538. doi: 10.18203/2394-6040.ijcmph20190054
19. De Godoy JMP, De Godoy HJP, Godoy MFG. Evaluation of Impedance and Reactance in the Intensive Treatment of Lymphoedema. Int J Sci Res. 2019; 7: 30-33. doi: 10.7860/JCDR/2019/40158.12806
20. Bjerum CM, Ouattara AF, Aboulaye M, Kouadio O, Marius VK, Andersen BJ, Weil GJ, Koudou BG, King CL. Efficacy and Safety of a Single Dose of Ivermectin, Diethylcarbamazine, and Albendazole for Treatment of Lymphatic Filariasis in Côte d'Ivoire: An Open-label Randomized Controlled Trial. Clin Infect Dis. 2020; 71: e68-e75. doi: 10.1093/cid/ciz1050

1. De Britto L, Kamaraj P, Vijayalakshmi G, Das LK, Krishnakumari A, Anbusivam S, Supriya VK, Yuvaraj J. Clinical Benefits of Supervised Home Care of the Morbidity Management and Disability Prevention Under National Filariasis Elimination Programme. Lymphat Res Biol. 2020; 18: 517-525. doi: 10.1089/lrb.2019.0058
2. Douglass J, Hailekiros F, Martindale S, Mableson H, Seife F, Bishaw T, Nigussie M, Meribo K, Tamiru M, Agidew G, Kim S, Betts H, Taylor M, Kelly-Hope L. Addition of Lymphatic Stimulating Self-Care Practices Reduces Acute Attacks among People Affected by Moderate and Severe Lower-Limb Lymphedema in Ethiopia, a Cluster Randomized Controlled Trial. J Clin Med. 2020; 9: 4077. doi: 10.3390/jcm9124077
3. Dubray CL, Sircar AD, Beau de Rochars VM, Bogus J, Direny AN, Ernest JR, Fayette CR, Goss CW, Hast M, O'Brian K, Pavilus GE, Sabin DF, Wiegand RE, Weil GJ, Lemoine JF. Safety and efficacy of co-administered diethylcarbamazine, albendazole and ivermectin during mass drug administration for lymphatic filariasis in Haiti: Results from a two-armed, open-label, cluster-randomized, community study. PLoS Negl Trop Dis. 2020; 14: e0008298. doi: 10.1371/journal.pntd.0008298
4. Pion SDS, Chesnais CB, Awaca-Uvon NP, Vlaminck J, Abdou A, Kunyu-Shako B, Kuyangisa Simuna G, Tambwe JP, Weil GJ, Boussinesq M. The impact of four years of semiannual treatments with albendazole alone on lymphatic filariasis and soil-transmitted helminth infections: A community-based study in the Democratic Republic of the Congo. PLoS Negl Trop Dis. 2020; 14: e0008322. doi: 10.1371/journal.pntd.0008322
5. Shriram AN, Premkumar A, Krishnamoorthy K, De A, Paul SK, Subramanian S, Vijayachari P, Jambulingam P. Elimination of diurnally sub-periodic *Wuchereria bancrofti* in Andaman and Nicobar Islands, India, using mass DEC-fortified salt as a supplementary intervention to MDA. Parasitol Res. 2020; 119: 1467-1483. doi: 10.1007/s00436-020-06659-7
6. Mathiarasan L, Das LK, Krishnakumari A. Assessment of the Impact of Morbidity Management and Disability Prevention for Lymphatic Filariasis on the Disease Burden in Villupuram District of Tamil Nadu, India. Indian J Community Med. 2021; 46: 657-661. doi: 10.4103/ijcm.IJCM_12_21
7. Beyene AD, Kebede F, Mammo BM, Negash BK, Mihret A, Abetew S, Oucha AK, Alene S, Backers S, Mante S, Sifri Z, Brady M, McPherson S. The implementation and impact of a pilot hydrocele surgery camp for LF-endemic communities in Ethiopia. PLoS Negl Trop Dis. 2021; 15: e0009403. doi: 10.1371/journal.pntd.0009403
8. Graves PM, Sheridan S, Scott J, Amosa-Lei Sam F, Naseri T, Thomsen R, King CL, Lau CL. Triple-Drug Treatment Is Effective for Lymphatic Filariasis Microfilaria Clearance in Samoa. Trop Med Infect Dis. 2021; 6: 44. doi: 10.3390/tropicalmed6020044
9. Hardy M, Samuela J, Kama M, Tuicakau M, Romani L, Whitfeld MJ, King CL, Weil GJ, Grobler AC, Robinson LJ, Kaldor JM, Steer AC. Individual Efficacy and Community Impact of Ivermectin, Diethylcarbamazine, and Albendazole Mass Drug Administration for Lymphatic Filariasis Control in Fiji: A Cluster Randomized Trial. Clin Infect Dis. 2021; 73: 994-1002. doi: 10.1093/cid/ciab202
10. Jambulingam P, Kuttiatt VS, Krishnamoorthy K, Subramanian S, Srividya A, Raju HKK, Rahi M, Somani RK, Suryaprakash MK, Dwivedi GP, Weil GJ. An open label, block randomized, community study of the safety and efficacy of co-administered ivermectin, diethylcarbamazine plus albendazole vs. diethylcarbamazine plus albendazole for lymphatic filariasis in India. PLoS Negl Trop Dis. 2021; 15: e0009069. doi: 10.1371/journal.pntd.0009069
11. Ouattara AF, Bjerum CM, Aboulaye M, Kouadio O, Marius VK, Andersen B, Lew D, Goss CW, Weil GJ, Koudou BG, King CL. Semiannual Treatment of Albendazole Alone is Efficacious for Treatment of Lymphatic Filariasis: A Randomized Open-label Trial in Cote d'Ivoire. Clin Infect Dis. 2022; 74: 2200-2208. doi: 10.1093/cid/ciab194
12. Supali T, Djuardi Y, Christian M, Iskandar E, Alfian R, Maylasari R, Destani Y, Lomiga A, Minggu D, Lew D, Bogus J, Weil GJ, Fischer PU. An open label, randomized clinical trial to compare the tolerability and efficacy of ivermectin plus diethylcarbamazine and albendazole vs. diethylcarbamazine plus albendazole for treatment of brugian filariasis in Indonesia. PLoS Negl Trop Dis. 2021; 15: e0009294. doi: 10.1371/journal.pntd.0009294
13. Pion SDS, Chesnais CB, Weil GJ, Louya F, Boussinesq M, Missamou F. Impact of Semi-Annual Albendazole on Lymphatic Filariasis and Soil-Transmitted Helminth Infection: Parasitological Assessment after 14 Rounds of Community Treatment. Am J Trop Med Hyg. 2021; 106: 729-731. doi: 10.4269/ajtmh.21-0731
14. Tavul L, Laman M, Howard C, Kotty B, Samuel A, Bjerum C, O'Brian K, Kumai S, Amuga M, Lorry L, Kerry Z, Kualawi M, Karl S, Makita L, John LN, Bieb S, Wangi J, Weil GJ, Goss CW, Tisch DJ, Pomat W, King CL, Robinson LJ. Safety and efficacy of mass drug administration with a single-dose triple-drug regimen of albendazole + diethylcarbamazine + ivermectin for lymphatic filariasis in Papua New Guinea: An open-label, cluster-randomised trial. PLoS Negl Trop Dis. 2022; 16: e0010096. doi: 10.1371/journal.pntd.0010096
15. Djuardi Y, Jannah IF, Supali T. IgG4 antibodies against Bm14 as an evaluation tool of mass drug administration in a co-endemic area of *Brugia timori* and *Wuchereria bancrofti*. Acta Trop. 2022; 227: 106278. doi: 10.1016/j.actatropica.2021.106278
16. De Santis G, Saxena B, Starnoni M, Pappalardo M, Jacob V. Efficacy of Vascularized Submental Lymph Node Transfer with Decongestive Therapy and Antibiotics for Early-Stage Lower Limb Filarial Lymphedema. Plast Reconstr Surg. 2023; 151: 850e-856e. doi: 10.1097/PRS.0000000000010046.
