# Supplementary material for: The lymphatic filariasis treatment study landscape: A systematic review of study characteristics and the case for an individual participant data platform
Source: PLoS Negl Trop Dis. 2024 Jan 16;18(1):e0011882. doi: 10.1371/journal.pntd.0011882 (PMC10817204; doi:10.1371/journal.pntd.0011882)
Supplement: Attachment — Submitted filename: Responses to Reviewers.docx [file pntd.0011882.s005.docx]

# Responses to Reviewers’ comments

## Reviewer 1

### Reviewer’s comment 1.1

The methods were clearly stated from the outset and were appropriate to achieve the stated aim of the review. The initial collection was clearly very extensive as shown by the very large attrition rate when removing duplicates from the large number of publication depositories. Overall no concerns

### Authors’ response 1.1.

We thank the Reviewer for the encouraging words and appreciation.

### Reviewer’s comment 1.2

The results are clearly and appropriately presented. The figures and tables are clear and provide additional information that assists in the understanding of the results.

### Authors’ response 1.2

We thank the Reviewer for the encouraging comments.

### Reviewer’s comment 1.3

The conclusions are clear and the limitations are adequately discussed. Since the main aim of this work was to identify material currently available to advance public health interventions in LF, these are covered in detail. Overall, this paper provides the benchmark for future work collection and curating individual patient data in LF and to plan prospective data collection. Overall, the aims of the study and publication of the material are achieved.

### Authors’ response 1.3

We thank the Reviewer for the appreciative comments.

### Reviewer’s comment 1.4

The paper is well written and easily understandable. it should be accepted without the need for additional modification.

### Authors’ response 1.4

We thank the Reviewer for this very supportive comment.

### Reviewer’s comment 1.5

The output of this work has considerable importance and has the potential to answer many of the questions that have dogged the LF community. The identification of areas where data is lacking, for example in MMDP studies and in treatment data from Africa, is an important step, although those in the field would be able to identify these from their own experience. The key step is to identify what data is often missing from data sets and ensure that these are collected in the future. The need to develop guidelines for collection and presentation is essential and probably reaches across all studies and diseases. It came as a surprise that even simple things like age and sex are an issue in these data sets. It is impressive that the data cut off for analysis was as late as May this year, meaning that an enormous amount of work in writing has been achieved in a very short space of time.

### Authors’ response 1.5

We thank the Reviewer for the appreciative and thoughtful comments. We agree that one of the important findings of the paper is in identifying geographical and other gaps in the LF data landscape. The Reviewer also correctly highlights that simple and ubiquitous variables such as age and sex are frequently not reported in sufficient detail to evaluate the demographic breakdown of individual participant data. This point is highlighted in the Discussion of the manuscript.

“The difficulty in estimation using reported information is particularly apparent when trying to quantify IPD associated with individual-level variables. For example, most studies provided only age eligibility criteria of participants, so we were not able to estimate a detailed demographic breakdown of IPD. Similarly, for sex, only 47 of the 138 studies reported sufficient information to estimate IPD for males and females to reach conclusions on gender representativeness, although notably, we identified 19 studies that included male only participants and no female-only studies.”

## Reviewer 2

### Reviewer’s comment 2.1

The authors have done a systematic review of literature to estimate the abundance of Individual participant data (IPD)on pre- and post-intervention indicators of LF infection and/or LF morbidity and assess the feasibility of building a global data repository. This is also aimed at developing a better strategy for elimination of LF. The study design is appropriate but the real data the authors could make available is small compared to the abundance of literature. Due to problems with the research studies itself the authors could select only 147 full text articles for analysis. I would like to congratulate the authors for the commendable and systematic efforts to identify the eligible articles. There are no ethical or regulatory concerns. The statistical methods used are appropriate.

### Authors’ response 2.1

We thank the Reviewer for the appreciative comments.

### Reviewer’s comment 2.2

The analysis is presented as per the plan itself. The authors were looking for the IPD regarding management of infection (preventive chemotherapy) and that of management of disease, the morbidity management and disability prevention. The tables and figures explain well the results of analysis. The results are well presented.

### Authors’ response 2.2

We thank the Reviewer for these encouraging comments.

### Reviewer’s comment 2.3

The authors had limitations to obtain the IPD on morbidity management and disability prevention. The Global Program to Eliminate LF and its preventive chemotherapy is a well-designed public health program and lots of data have been already generated on that. But the other strategy of MMDP has not been taken up by all the countries and the data that could be made available in this study is minimal only. This is the limitation of this study also.

### Authors’ response 2.3

We thank the Reviewer for this feedback. We agree with the Reviewer that, while preventive chemotherapy has been successfully implemented in most endemic countries, the other pillar of LF control, namely MMDP, is yet to be fully implemented, excepting a few highly endemic areas. Our analysis found that there are very few cohort-based studies that assess the impact of various MMDP strategies on disease outcomes. We have emphasised this in the manuscript, and we hope that this will trigger interest among researchers to initiate new MMDP studies. For example, we write in the Abstract and Discussion:

“New studies using more standardized approaches should be initiated to address the scarcity and inconsistency of data on morbidity management.”

“Approximately 20% of IPD comprises pre- and post-intervention morbidity indicators, revealing a relative paucity of information on the efficacy of morbidity management approaches, despite MMDP being one of the pillars of the LF elimination program.”

“Further and more standardised studies are urgently needed in this domain and should include post-surgery follow-up of participants to quantify relapse rates and the occurrence of secondary infections.”

### Reviewer’s comment 2.4

Here the goal of the authors is to develop a global data repository for LF infection and LF morbidity. For this, the authors have resorted to doing the systematic review of the available studies. The massive data available on LF elimination data- the preventive chemotherapy is the Global Health Observatory for Lymphatic Filariasis. Here the data of the number of people to whom preventive chemotherapy was given, percentage consumption in the country and all details are available. But authors have not mentioned about this at all. As a reviewer, I would like to have an opinion from the authors who have done lot of work, on how this repository will help them or not help them to achieve their goals of this study. Data on MMDP also will be available but may not be full proof. The authors may be encouraged to get details of global health observatory LF and may be added here in this manuscript how that would help to develop a repository important for LF elimination. I am not in a position to give recommendation but once this is done this can be considered for acceptance.

### Authors’ response 2.4

We thank the Reviewer for the comment. We are aware of the Global Health Observatory for LF, which provides data on preventive chemotherapy for most endemic countries. The data in the repository are aggregated and provide information on the population covered by MDA, the type of MDA (double or triple drug regimens) and the year MDA was distributed. However, it does not contain any individual level data and hence it was not considered in the review. We clarify in the revised manuscript the distinction between individual participant data repositories and the more common population-level epidemiological databases such as the Global Health Observatory

“The Infectious Diseases Data Observatory (IDDO) is an example of an established model based on a repository infrastructure that gathers dispersed and disparate IPD from scattered studies to create standardised datasets that allow researchers to address critical research gaps in a number of infectious diseases, beginning with the Worldwide Antimalarial Resistance Network (WWARN) in 2009 and extending to NTDs, including Chagas disease [27], visceral leishmaniasis [28], schistosomiasis and the soil-transmitted helminthiases [29], and emerging infections including Ebola and SARs-CoV-2 [30]. These platforms focus on the collation, curation and management of information-rich IPD, making them distinct from other population-level epidemiological data repositories such as the Expanded Special Project for Elimination of Neglected Tropical Diseases (ESPEN) portal (https://espen.afro.who.int) and the WHO’s Global Health Observatory (https://www.who.int/data/gho).”

### Reviewer’s comment 2.5

All the authors have done lot of committed work from the designing, collection of data, analysis and writing the manuscript. Similarly, lots of efforts have gone into writing this manuscript also. So this is to be considered as a good write up in this context which could become an inspiration to other researchers. So with revisions this may be accepted. The authors have also explained the limitations of the study.

### Authors’ response 2.5

We are sincerely grateful to the Reviewer for these kind and appreciative comments.
